# Supplementary material for: A multi-dimensional assessment of the 2021 mouse plague in New South Wales, Australia: Economic impacts and policy responses
Source: PLoS One. 2026 Apr 21;21(4):e0338405. doi: 10.1371/journal.pone.0338405 (PMC13098966; doi:10.1371/journal.pone.0338405)
Supplement: Attachment — Submitted filename: Response to Reviewers.docx [file pone.0338405.s004.docx]

Journal Requirements:

When submitting your revision, we need you to address these additional requirements.

1. Please ensure that your manuscript meets PLOS ONE's style requirements, including those for file naming. The PLOS ONE style templates can be found at <https://journals.plos.org/plosone/s/file?id=wjVg/PLOSOne_formatting_sample_main_body.pdf> and <https://journals.plos.org/plosone/s/file?id=ba62/PLOSOne_formatting_sample_title_authors_affiliations.pdf>

We have now submitted the revised manuscript as ‘Revised Manuscript with Tracked Changes’, the manuscript as ‘Manuscript’, the supporting information as ‘Supporting File’ with legend, and the response to reviewer comments as ‘Response to Reviewers’.

We have edited the affiliations – please see L4-15 of the manuscript.

The revised manuscript (submitted as ‘Manuscript’) has now been divided into Abstract (bold and font size 18 – see L16), Introduction (bold and font size18 – see L30), Materials and method (bold and font size 18 – see L102), Results and discussion (bold and font size 18 -see L358), Conclusion (bold and font size 18 – see L653), Acknowledgements (bold and font size 18 – see 665), and References (bold and font size 18 – see L671). The sub-headings i.e., headings two and three, which are now only in the Materials and methods section are now in bold and fonts 16 and 14, respectively – please see L103, L122, L136, L141, L213, L214, L300, and L313.

The intext figures have now been revised to ‘Fig’ and titled according to the journal requirements. Please see L134-135, L470-471, L472-476, and L496-497. All the figures have been checked with NAAS, and they meet the journal requirement.

The intext references are now numbered.

We have checked all the references and confirm that none is missing. The references have now been revised according to the journal style – see L673-839.

2. Please include your full ethics statement in the ‘Methods’ section of your manuscript file. In your statement, please include the full name of the IRB or ethics committee who approved or waived your study, as well as whether or not you obtained informed written or verbal consent. If consent was waived for your study, please include this information in your statement as well.

A full ethics statement has now been included in the ‘Materials and methods’ section -please see L207-212.

3. We note that your Data Availability Statement is currently as follows: All relevant data are within the manuscript and its Supporting Information files.

Please confirm at this time whether or not your submission contains all raw data required to replicate the results of your study. Authors must share the “minimal data set” for their submission. PLOS defines the minimal data set to consist of the data required to replicate all study findings reported in the article, as well as related metadata and methods (<https://journals.plos.org/plosone/s/data-availability#loc-minimal-data-set-definition>).

For example, authors should submit the following data:

- The values behind the means, standard deviations and other measures reported;
- The values used to build graphs;
- The points extracted from images for analysis.

Authors do not need to submit their entire data set if only a portion of the data was used in the reported study.

If your submission does not contain these data, please either upload them as Supporting Information files or deposit them to a stable, public repository and provide us with the relevant URLs, DOIs, or accession numbers. For a list of recommended repositories, please see <https://journals.plos.org/plosone/s/recommended-repositories>.

If there are ethical or legal restrictions on sharing a de-identified data set, please explain them in detail (e.g., data contain potentially sensitive information, data are owned by a third-party organization, etc.) and who has imposed them (e.g., an ethics committee). Please also provide contact information for a data access committee, ethics committee, or other institutional body to which data requests may be sent. If data are owned by a third party, please indicate how others may request data access.

We have submitted the de-identified data used for the statistics (e.g., means, maximum, and minimum) and the graphs as supporting file – please see S1_File. This is almost the entire dataset used in the study and has been arranged according to the entity affected (i.e., household, farm, and business/facility) making it easy to follow and replicate. Please note that the data for Fig 4 is within the text and the reference provided. We confirm that the raw data used to determine the results are within the manuscript text and the supporting file.

4. We note that the grant information you provided in the ‘Funding Information’ and ‘Financial Disclosure’ sections do not match.

When you resubmit, please ensure that you provide the correct grant numbers for the awards you received for your study in the ‘Funding Information’ section.

Our sincere apologies and thanks for noticing this. We have now included the following statement in our cover letter: ‘This research was funded by the NSW Department of Primary Industries and Regional Development. The funders had no role in study design, data collection and analysis, decision to publish, or preparation of the manuscript.’

5. Thank you for stating the following financial disclosure:
The study was funded by the Department of Regional NSW “Research and monitoring

program to support more effective management of mice in NSW” with support from

CSIRO Health & Biosecurity.

Please state what role the funders took in the study. If the funders had no role, please state: "The funders had no role in study design, data collection and analysis, decision to publish, or preparation of the manuscript."
If this statement is not correct you must amend it as needed.
Please include this amended Role of Funder statement in your cover letter; we will change the online submission form on your behalf.

Our sincere apology for this error. We confirm that the funders had role in the research. Consequently, we have now revised and included our funding statement in the cover letter, and it reads as follows:

‘This research was funded by the NSW Department of Primary Industries and Regional Development. The funders had no role in study design, data collection and analysis, decision to publish, or preparation of the manuscript.’

6. Thank you for stating the following in the Acknowledgments Section of your manuscript:
The authors acknowledge the NSW community for participating in the study and providing the data that underpinned this research. The authors acknowledge NSW Department of Primary Industries and the Grains Research & Development Corporation for their guidance and support. We also thank the online research provider for assisting in data collection, Dr. Loechel Barton for evaluating the questionnaire, and various community groups and research facilitators for distributing the survey link.

We note that you have provided funding information that is not currently declared in your Funding Statement. However, funding information should not appear in the Acknowledgments section or other areas of your manuscript. We will only publish funding information present in the Funding Statement section of the online submission form.
Please remove any funding-related text from the manuscript and let us know how you would like to update your Funding Statement. Currently, your Funding Statement reads as follows:
The study was funded by the Department of Regional NSW “Research and monitoring

program to support more effective management of mice in NSW” with support from

CSIRO Health & Biosecurity.

Please include your amended statements within your cover letter; we will change the online submission form on your behalf.

Our sincere apologies. We have deleted the acknowledgement of the funder – please see L666-669 in the manuscript. We have equally provided a revised funding disclosure in the cover letter, and it reads as follows:

This research was funded by the NSW Department of Primary Industries and Regional Development. The funders had no role in study design, data collection and analysis, decision to publish, or preparation of the manuscript.

7. Please amend your list of authors on the manuscript to ensure that each author is linked to an affiliation. Authors’ affiliations should reflect the institution where the work was done (if authors moved subsequently, you can also list the new affiliation stating “current affiliation:….” as necessary).

We have now amended the list of authors with each other having their own affiliation – please see L4-10.

8. [We note that Figure 3 in your submission contain [map/satellite] images which may be copyrighted. All PLOS content is published under the Creative Commons Attribution License (CC BY 4.0), which means that the manuscript, images, and Supporting Information files will be freely available online, and any third party is permitted to access, download, copy, distribute, and use these materials in any way, even commercially, with proper attribution. For these reasons, we cannot publish previously copyrighted maps or satellite images created using proprietary data, such as Google software (Google Maps, Street View, and Earth). For more information, see our copyright guidelines: http://journals.plos.org/plosone/s/licenses-and-copyright.

We require you to either (1) present written permission from the copyright holder to publish these figures specifically under the CC BY 4.0 license, or (2) remove the figures from your submission:

a. You may seek permission from the original copyright holder of Figure 3 to publish the content specifically under the CC BY 4.0 license.

We recommend that you contact the original copyright holder with the Content Permission Form (http://journals.plos.org/plosone/s/file?id=7c09/content-permission-form.pdf) and the following text:
“I request permission for the open-access journal PLOS ONE to publish XXX under the Creative Commons Attribution License (CCAL) CC BY 4.0 (http://creativecommons.org/licenses/by/4.0/). Please be aware that this license allows unrestricted use and distribution, even commercially, by third parties. Please reply and provide explicit written permission to publish XXX under a CC BY license and complete the attached form.”

Please upload the completed Content Permission Form or other proof of granted permissions as an "Other" file with your submission.

In the figure caption of the copyrighted figure, please include the following text: “Reprinted from [ref] under a CC BY license, with permission from [name of publisher], original copyright [original copyright year].”

b. If you are unable to obtain permission from the original copyright holder to publish these figures under the CC BY 4.0 license or if the copyright holder’s requirements are incompatible with the CC BY 4.0 license, please either i) remove the figure or ii) supply a replacement figure that complies with the CC BY 4.0 license. Please check copyright information on all replacement figures and update the figure caption with source information. If applicable, please specify in the figure caption text when a figure is similar but not identical to the original image and is therefore for illustrative purposes only.
The following resources for replacing copyrighted map figures may be helpful:

USGS National Map Viewer (public domain): http://viewer.nationalmap.gov/viewer/
The Gateway to Astronaut Photography of Earth (public domain): http://eol.jsc.nasa.gov/sseop/clickmap/
Maps at the CIA (public domain): https://www.cia.gov/library/publications/the-world-factbook/index.html and https://www.cia.gov/library/publications/cia-maps-publications/index.html
NASA Earth Observatory (public domain): http://earthobservatory.nasa.gov/
Landsat: http://landsat.visibleearth.nasa.gov/
USGS EROS (Earth Resources Observatory and Science (EROS) Center) (public domain): http://eros.usgs.gov/#
Natural Earth (public domain): http://www.naturalearthdata.com/](http://track.editorialmanager.com/CL0/We%2520note%2520that%2520Figure%25203%2520in%2520your%2520submission%2520contain%2520%255bmap%2Fsatellite%255d%2520images%2520which%2520may%2520be%2520copyrighted.%2520All%2520PLOS%2520content%2520is%2520published%2520under%2520the%2520Creative%2520Commons%2520Attribution%2520License%2520(CC%2520BY%25204.0),%2520which%2520means%2520that%2520the%2520manuscript,%2520images,%2520and%2520Supporting%2520Information%2520files%2520will%2520be%2520freely%2520available%2520online,%2520and%2520any%2520third%2520party%2520is%2520permitted%2520to%2520access,%2520download,%2520copy,%2520distribute,%2520and%2520use%2520these%2520materials%2520in%2520any%2520way,%2520even%2520commercially,%2520with%2520proper%2520attribution.%2520For%2520these%2520reasons,%2520we%2520cannot%2520publish%2520previously%2520copyrighted%2520maps%2520or%2520satellite%2520images%2520created%2520using%2520proprietary%2520data,%2520such%2520as%2520Google%2520software%2520(Google%2520Maps,%2520Street%2520View,%2520and%2520Earth).%2520For%2520more%2520information,%2520see%2520our%2520copyright%2520guidelines:%2520http:%2Fjournals.plos.org%2Fplosone%2Fs%2Flicenses-and-copyright.%250b%250bWe%2520require%2520you%2520to%2520either%2520(1)%2520present%2520written%2520permission%2520from%2520the%2520copyright%2520holder%2520to%2520publish%2520these%2520figures%2520specifically%2520under%2520the%2520CC%2520BY%25204.0%2520license,%2520or%2520(2)%2520remove%2520the%2520figures%2520from%2520your%2520submission:%250b%250ba.%2520You%2520may%2520seek%2520permission%2520from%2520the%2520original%2520copyright%2520holder%2520of%2520Figure%25203%2520to%2520publish%2520the%2520content%2520specifically%2520under%2520the%2520CC%2520BY%25204.0%2520license.%2520%250b%250bWe%2520recommend%2520that%2520you%2520contact%2520the%2520original%2520copyright%2520holder%2520with%2520the%2520Content%2520Permission%2520Form%2520(http:%2Fjournals.plos.org%2Fplosone%2Fs%2Ffile%3Fid=7c09%2Fcontent-permission-form.pdf)%2520and%2520the%2520following%2520text:%250b%E2%80%9CI%2520request%2520permission%2520for%2520the%2520open-access%2520journal%2520PLOS%2520ONE%2520to%2520publish%2520XXX%2520under%2520the%2520Creative%2520Commons%2520Attribution%2520License%2520(CCAL)%2520CC%2520BY%25204.0%2520(http:%2F%2Fcreativecommons.org%2Flicenses%2Fby%2F4.0%2F).%2520Please%2520be%2520aware%2520that%2520this%2520license%2520allows%2520unrestricted%2520use%2520and%2520distribution,%2520even%2520commercially,%2520by%2520third%2520parties.%2520Please%2520reply%2520and%2520provide%2520explicit%2520written%2520permission%2520to%2520publish%2520XXX%2520under%2520a%2520CC%2520BY%2520license%2520and%2520complete%2520the%2520attached%2520form.%E2%80%9D%250b%250bPlease%2520upload%2520the%2520completed%2520Content%2520Permission%2520Form%2520or%2520other%2520proof%2520of%2520granted%2520permissions%2520as%2520an%2520%2522Other%2522%2520file%2520with%2520your%2520submission.%250b%250bIn%2520the%2520figure%2520caption%2520of%2520the%2520copyrighted%2520figure,%2520please%2520include%2520the%2520following%2520text:%2520%E2%80%9CReprinted%2520from%2520%255bref%255d%2520under%2520a%2520CC%2520BY%2520license,%2520with%2520permission%2520from%2520%255bname%2520of%2520publisher%255d,%2520original%2520copyright%2520%255boriginal%2520copyright%2520year%255d.%E2%80%9D%250b%250bb.%2520If%2520you%2520are%2520unable%2520to%2520obtain%2520permiss/1/010f019b9149d8c7-b506eb11-a4fb-4a7f-a8b4-d67ca17ffb99-000000/H_jwzXhgmVN-WTTZkrukcMdXZeGUpU6zWeXFJ8BnPao=244)

Figure 3 was produced using ArcMap 10.8.2 (ESRI) as the cartographic software only. No ESRI basemaps, imagery, or proprietary ESRI data were used. However, the local government area (LGA) administrative boundaries and the town point datasets were obtained from Geoscape Australia and unfortunately, we did not provide the attribution. We have now provided the attribution for both the software and dataset used to create Fig 3 (L472-476). The dataset attribution follows the format recommended by Geoscape Australia - please see the Geoscape Australia reference in L795-797 to find the link.

Also, we have confirmed with the Department of Industry, Science and Resources (DISR) who are the original copyright (CC BY 4.0.) holders– please see the email confirmation titled ‘Copyright confirmation’ that we’ve submitted – that the dataset conforms with the CC BY 4.0 requirements and can be used/distributed by journals and any other third party without any restrictions. Please note that Geoscape Australia manages the datasets on behalf of DISR and that DISR does not provide copyright permissions to individuals as they have stated in their email correspondence.

9. If the reviewer comments include a recommendation to cite specific previously published works, please review and evaluate these publications to determine whether they are relevant and should be cited. There is no requirement to cite these works unless the editor has indicated otherwise.

Fortunately, none of the reviewers recommended any published work.
10. Please review your reference list to ensure that it is complete and correct. If you have cited papers that have been retracted, please include the rationale for doing so in the manuscript text, or remove these references and replace them with relevant current references. Any changes to the reference list should be mentioned in the rebuttal letter that accompanies your revised manuscript. If you need to cite a retracted article, indicate the article’s retracted status in the References list and also include a citation and full reference for the retraction notice.

We have checked the reference list, and we confirm that it is complete and correct. Also, we have now reviewed the reference list and made sure that the references follow the PLoS One style – please see L672-837.

No retracted reference/publication has been cited in our study.

We have made the following changes to the reference list:

- We have added Geoscape Australia in the reference (L795-797) as part of the attribution of the dataset we used to create Fig 3.
- We have included Heenan et al. 2003 (L830-832) and Hoare et al. 2006 (L833-834) to support our statements on the impact of chemical mouse baits on the environment, animals and human health. One of the reviewers suggested that we mention why the farmers were restricting their use of chemical mouse baits hence the inclusion of the two references.

Additional Editor Comments:

Dear authors,

Thank you for resubmitting your manuscript to PLOS ONE. Reviewers have recommended reconsideration of the draft following minor revisions. Please, consider the following during revision.

1. The current title is repetitive. A strong title usually answers one MAIN QUESTION, and rarely three. That is:

(a) ‘Economic impact’ already implies costs

(b) ‘Quantifying cost’ restates that same idea

(c) The ‘policy variables’ and ‘control options’ to my understanding relates to intervention and/or management.

Two non-repetitive alternative titles I would suggest are:

A multi-dimensional assessment of the 2021 mouse plague in New South Wales, Australia: Economic impacts and policy responses

Economic impacts and control strategies of the 2021 mouse plague in New South Wales, Australia

We agree with this suggestion. We have now changed the title of the manuscript to ‘A multi-dimensional assessment of the 2021 mouse plague in New South Wales, Australia: Economic impacts and policy responses’ – please see L1-3.

2. Please format the intext citations and references to the journal style.

Thank you for this observation. We have now revised the intext citation using numbering as well as the references to match the journal style/requirements (L672-837).

3. I have added some comments and suggestions in the attached MS file which could improve manuscript clarity.

Thank you for your valuable comments and suggestions. We have incorporated all the suggestions into the revised manuscript.

We have made the following changes in the abstract section of the manuscript as suggested:

- We have replaced ‘our’ with the ‘present study’ (L19).
- We have revised ‘economic impact’ to ‘economic impacts” (L19).
- We have revised ‘….crop farmers to reduce crop yield losses’ to ‘….farmers to minimize avoidable crop yield losses’ (L22-23).
- We have included ‘2021’ in L23.
- We have rounded off the ‘A$ 100,620,695’ to ‘A$ 100.62’ (L23-24).
- We have revised ‘The study also revealed type of farming’ to ‘It was also revealed that the type of farming’ (L24-25).
- We have revised ‘….each type used’ to ‘….each rodenticide when…’ (L27).
- We have included ‘how’ in L28.
- We have included ‘of mouse plagues’ in L29.
- We have replaced ‘related’ with ‘associated’ in L29.

We have made the following changes in the rest of the manuscript as suggested:

- We have revised ‘impact’ to ‘impacts’ (L33 and L37) and included the word ‘can’ (L33).
- We have removed the words ‘grain and other’ (L36).
- We have changed ‘is’ to ‘are’ (L38).
- We have removed all the subheadings in the introduction section (L31-101).
- We have replaced ‘After the 1970 mouse plague’ with ‘In 1980’ (L42) and included ‘another’ (L42).
- We have removed ‘in Australia’ (L47) and replaced ‘with the plague spreading across’ with ‘affecting’ (L48).
- The sentence ‘To exacerbate the situation, there was a simultaneous’ has been replaced with ‘This coincided with the’ (L51). Also, we have deleted ‘in NSW and the rest of Australia’ (L51-52) and added ‘NSW’ (L52) as suggested.
- We have revised ‘wild house mouse’ to ‘wild mouse’ as suggested (L56)
- ‘…a 20% reduction in egg production’ has now been revised to ‘….caused a 20% reduction in poultry egg production’ as suggested (L65-66).
- We have revised the sentence ‘….1980 and 1984 mouse plague in the state of Victoria was’ to ‘….1980 and 1984 mouse plagues in the state of Victoria were…’ (L66-67) and deleted ‘average of A$ 17.5 million’ (L67).
- We have deleted ‘Additionally, according to Caughley et al. (1994)’ and replaced it with ‘Further’. (L68). Also, we have included the Caughely et al. 1994 reference (as a number) at the end of the sentence (L70).
- The sentence describing the chemical methods in the introduction section has now been revised and divided into two to improve clarity (L73-76). We have also added ‘anticoagulants’ as another example for completion (L74).
- ‘…could apply..’ has now been revised to a past tense (‘..applied..’) as suggested (L81).
- The sentence ‘Studies on the economic impact of mouse plagues are limited despite its continuous occurrence’ has now been deleted (L83).
- The typo ‘plaque’ has been revised to ‘plague’ (L100).
- ‘is’ has been revised to ‘are’ (L139).
- Table 1 has now been revised to include n (%) as suggested (L377)
- The first two rows in Table 4 have now been combined to have only one citation as suggested (L499).
- We have revised ‘crop farmer’ to ‘crop farmers’ (L553).
- We have revised ‘the Caughley et al. (1994) report was’ to ‘that of [7] is’ (L562).
- We have revise ‘economic impact’ to ‘economic impacts’ (L577).

[Note: HTML markup is below. Please do not edit.]

Reviewers' comments:

Reviewer's Responses to Questions

**Comments to the Author**

1. Is the manuscript technically sound, and do the data support the conclusions?

The manuscript must describe a technically sound piece of scientific research with data that supports the conclusions. Experiments must have been conducted rigorously, with appropriate controls, replication, and sample sizes. The conclusions must be drawn appropriately based on the data presented.

Reviewer #1: Yes

Reviewer #2: Yes

2. Has the statistical analysis been performed appropriately and rigorously?

Reviewer #1: Yes

Reviewer #2: Yes

3. Have the authors made all data underlying the findings in their manuscript fully available?

The [PLOS Data policy](http://track.editorialmanager.com/CL0/http:%2F%2Fwww.plosone.org%2Fstatic%2Fpolicies.action%23sharing/1/010f019b9149d8c7-b506eb11-a4fb-4a7f-a8b4-d67ca17ffb99-000000/qcXAryDg7wNxQJveiONHnYAKbp4_JOApeJRBJqTfFUw=244) requires authors to make all data underlying the findings described in their manuscript fully available without restriction, with rare exception (please refer to the Data Availability Statement in the manuscript PDF file). The data should be provided as part of the manuscript or its supporting information, or deposited to a public repository. For example, in addition to summary statistics, the data points behind means, medians and variance measures should be available. If there are restrictions on publicly sharing data—e.g. participant privacy or use of data from a third party—those must be specified.

Reviewer #1: Yes

Reviewer #2: Yes

4. Is the manuscript presented in an intelligible fashion and written in standard English?

PLOS ONE does not copyedit accepted manuscripts, so the language in submitted articles must be clear, correct, and unambiguous. Any typographical or grammatical errors should be corrected at revision, so please note any specific errors here.

Reviewer #1: Yes

Reviewer #2: Yes

5. Review Comments to the Author

Please use the space provided to explain your answers to the questions above. You may also include additional comments for the author, including concerns about dual publication, research ethics, or publication ethics. (Please upload your review as an attachment if it exceeds 20,000 characters)

Reviewer #1: Well thought out and systematic analysis on the economic impact of mouse plague with findings, potential limitations clearly aritculated and appropriate implications for policy and practice outlined. A pleasure to read -- congratulations!

Thank you.

Reviewer #2: The manuscript titled ‘Multi-analysis of the 2021 mouse plague economic impact in New South Wales, Australia: Quantifying cost, policy variables, and efficient control options’ is interesting and need of the hour as it focuses on the agrarian impact of the 2021 mouse plague. It is disturbing to note that even after being severely impacted by the mouse plague, farmers themselves had to bear 67.10% of the total cost of loss. This result forms a valuable insight, seeking planned interventions from the policy front.

Thank you for your valuable comments.

1. Authors are advised to revise the figures and graphs, especially figure. 3, as figure texts are not legible.

Our apology if any of the figures were illegible. This could be because of the conversion of the figures which are in the TIFF format being printed as PDF for the reviewers. We have now revised the figures and used NAAS to ensure that they conform to the journal requirements. Fig 3 is much more legible as TIFF especially using zooming.

2. Please mention how all the farmers including cereals, pulses, vegetables, livestock etc are equally impacted by the mouse plague? sampling gave equal weightage to all farmers alike? Is it the ground reality .

Yes, all the various types of the farmers were equally impacted by the mouse plague, and this is the reality on the ground. We have now highlighted this in the results and discussion section (L569-576).

3. Kindly mention in the discussion section, why farmers were restricting/ limiting mouse control options as only the chemical used by crop farmers to reduce crop yield losses, and have they found it to be counterproductive or any negative impacts of it health of the produce or farmers.

The restriction of the use of the chemical options was because of the government safety regulation requirements and recommendations as they can have negative health impacts on users (e.g., farmers handling the mouse bait), other people, animals, and the environment. We have now highlighted this in the results and discussion section (L632-640).

4. grammatical/ typo errors have to be corrected.

We have corrected the typo errors. For example, we have revised ‘plaque’ to ‘plague’ (L100), and ‘Accommodation services was’ to ‘Accommodation services were’ (L443).

6. PLOS authors have the option to publish the peer review history of their article ([what does this mean?](https://track.editorialmanager.com/CL0/https:%2F%2Fjournals.plos.org%2Fplosone%2Fs%2Feditorial-and-peer-review-process%23loc-peer-review-history/1/010f019b9149d8c7-b506eb11-a4fb-4a7f-a8b4-d67ca17ffb99-000000/8W44WOy8dn7d__D2gH3rvJGg8whIyNaxXuSObS5Bbwc=244)). If published, this will include your full peer review and any attached files.


If you choose “no”, your identity will remain anonymous but your review may still be made public.


**Do you want your identity to be public for this peer review?** For information about this choice, including consent withdrawal, please see our [Privacy Policy](https://track.editorialmanager.com/CL0/https:%2F%2Fwww.plos.org%2Fprivacy-policy/1/010f019b9149d8c7-b506eb11-a4fb-4a7f-a8b4-d67ca17ffb99-000000/nIoeBJY_GOk-2_2kOm5_HS6pKATipT25_a-RjVV4wOQ=244).

Reviewer #1: No

Reviewer #2: **Yes:** Dhanya Punnoli

[NOTE: If reviewer comments were submitted as an attachment file, they will be attached to this email and accessible via the submission site. Please log into your account, locate the manuscript record, and check for the action link "View Attachments". If this link does not appear, there are no attachment files.]

We checked the attachment files and found the original manuscript with the Editor comments and edits. We have incorporated all the suggestions and edits from the Editor as indicated earlier in this response.

To ensure your figures meet our technical requirements, please review our figure guidelines: <https://journals.plos.org/plosone/s/figures>

You may also use PLOS’s free figure tool, NAAS, to help you prepare publication quality figures: <https://journals.plos.org/plosone/s/figures#loc-tools-for-figure-preparation>.

NAAS will assess whether your figures meet our technical requirements by comparing each figure against our figure specifications.

We have submitted all the figures to NAAS, and they now meet the journal requirement. Fig 1 was converted from PDF to TIFF using NAAS. Fig 2 and 4 were re-created using RStudio and saved as EPS files while maintaining the aspect ratios. They were then converted to TIFF using NAAS. Fig 3 was converted from PNG to TIFF using NAAS.
